# Cellular and structural insight into dynamin function during endocytic vesicle formation: a tale of 50 years of investigation

**DOI:** 10.1042/BSR20211227

**Published:** 2022-11-10

**Authors:** David Perrais

**Affiliations:** CNRS, Interdisciplinary Institute for Neuroscience, IINS, University of Bordeaux, UMR 5297, F-33000 Bordeaux, France

**Keywords:** clathrin, dynamin, endocytosis, live cell imaging

## Abstract

Dynamin is one of the major proteins involved in endocytosis. First identified 50 years ago in a genetic screen in *Drosophila melanogaster*, it has become a central player in many forms of endocytosis, such as clathrin-mediated endocytosis or synaptic vesicle endocytosis, as well as other important cellular processes such as actin remodelling. Decades of work using biochemical and structural studies, cell-free assays, live cell imaging, acute inhibition and genetic studies have led to important insights on its mode of action. Dynamin is a remarkable mechano-GTPase, which can do a lot to membranes on its own but which is, in cells, at the centre of a vast protein and lipid network and cannot work in isolation. This review summarizes the main features of dynamin structure and function and its central role in membrane remodelling events, and give an update on the latest results.

## Discovery of dynamin as an essential protein for endocytic vesicle scission – the first 30 years

**Figure 1:**
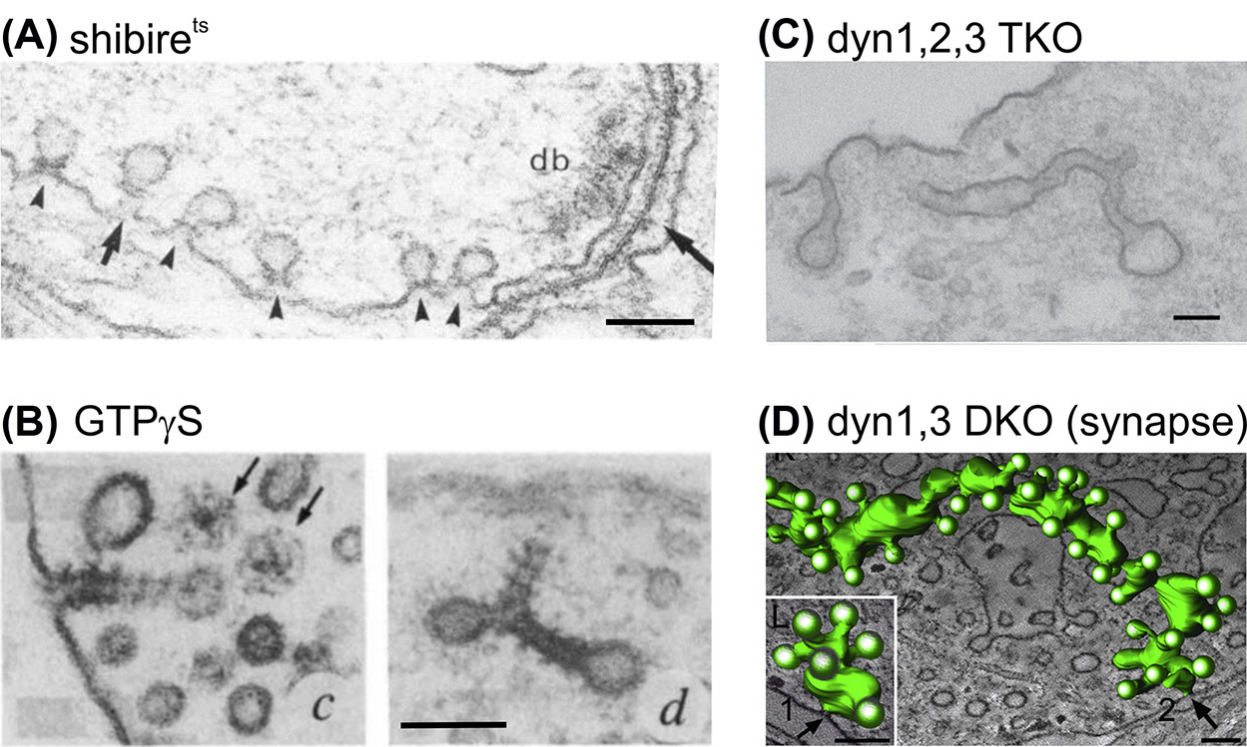
Electron micrographs illustrating the consequences of dynamin suppression or inactivation on endocytic vesicle formation (**A**) In the synaptic terminals of shibire^ts^ flies kept at paralysing temperature (29°C). Arrowheads point to collared endocytic pits. Note that the collar of the pit designated by the *short arrow* is seen as two parallel lines in this plane of sectioning. db, dense body; Long arrow, release site marked by the dense body (db); scale bar: 100 nm. Reprinted from [4]. Copyright 1989 Society for Neuroscience. (**B**) In lysed rat brain synaptosomes incubated with GTPγS, tubular invaginations are coated by regular spaced striations. Thin arrows indicate CCVs; scale bar: 100 nm. Reprinted from [7]. (**C**) In a dyn1,2,3 TKO fibroblast, CCPs connected to the plasma membrane by long, narrow tubular necks; scale bar: 100 nm. Reprinted from [8]. (**D**) In the synapse of a dyn1,3 DKO neuron, reconstructed endocytic structures originating from two sites (1 and 2, arrows), superimposed on individual electron tomography sections to illustrate the site of connection with the rest of the plasma membrane; scale bars: 100 nm. Reprinted from [9].

Endocytosis, the formation of vesicles from the plasma membrane, is a fundamental process occurring in all eukaryotic cells. Its essential role in organismal biology was exemplified with the isolation in 1972 of temperature-sensitive mutants of *Drosophila melanogaster* called shibire^ts^ (which means numb in Japanese) [1]. Mutant flies develop and behave normally when they are raised at a temperature of 22°C. However, increasing the temperature to 29°C leads to their progressive paralysis, which is complete within 2 min. If the ambient temperature is maintained at 29°C, mutant flies remain paralyzed for up to 12 h and their eventual death. Strikingly, bringing back the temperature to 22°C, surviving flies will promptly and fully recover within 3 min. The cellular basis of this remarkable phenotype was further studied: high temperature blocks synaptic transmission at the neuromuscular junction [2] and depletes presynaptic vesicles [3]. In addition to vesicle depletion, small invaginations of the presynaptic plasma membrane are observed (approximately 50 nm in diameter), which increase in number and size as the preparation is put back a permissive temperature [4]. Interestingly, the necks of the invaginations, approximately 20 nm in diameter, are often decorated with an electron dense ‘collar’, which can be seen in a minority of cases as two parallel lines, suggesting a helicoidal structure wrapped around the neck (Figure 1A). It was then hypothesized that this collar could correspond to the product of the *shibire* gene and would be responsible for an essential step of vesicle recycling through endocytosis. Shortly after, the *shibire* gene was identified and shown to be homologous to the mammalian gene dynamin, with approximately 80% of sequence homology throughout the coding sequence [5,6].

Dynamin was initially identified as a 100 kDa cytosolic protein co-purified with microtubules stabilized with Taxol in calf brain white matter extracts. Its name, dynamin, was chosen because, like the molecular motors kinesin or dynein, it is able to slide microtubules in an ATP-dependent manner [10] and has a microtubule-stimulated GTPase activity [11]. However, binding of microtubules seemed at odds with a direct role in endocytosis because microtubules rarely approach the plasma membrane. It was later shown that the binding to stable microtubules is relevant in cells during mitosis but not in cells in interphase [12]. In the human cell line HeLa, overexpression of a mutated dynamin, K44A, which lacks microtubule stimulated GTPase activity, blocks endocytosis, assayed with transferrin receptor (TfR) internalization. Moreover, dynamin K44A is located at clathrin-coated pits (CCPs), where clathrin-mediated endocytosis (CME) occurs [13,14]. Similarly, incubation of lysed rat brain nerve terminals with GTPγS, a non-hydrolysable form of GTP, led to the formation of tubular membrane invaginations with striated, electron dense material [7] (Figure 1B) reminiscent of the striated microtubules containing dynamin [10] or the collared pits in shibire^ts^ nerve terminals recovering from endocytosis block [4,15]. Indeed, these striated tubules are intensely immunoreactive for dynamin [7]. Endogenous wild-type dynamin is partitioned into a cytosolic fraction and a membrane fraction. Membrane-associated dynamin is primarily associated with CCPs on the plasma membrane [14]. Moreover, pure dynamin self assembles into ring-like structures in low salt conditions with an inner diameter of ∼25 nm, which corresponds to the outer diameter of microtubules or the neck of endocytic invaginations [16]. Therefore, dynamin was thought to initiate and/or stabilize the constriction of invaginations, and perhaps mediating scission through its GTPase activity. Finally, dynamin alone is able to bind to liposomes containing anionic phospholipids such as phosphatidylserine (PS) or phosphatidylinositol(4,5)diphosphate (PI(4,5)P_2_), which are enriched at the inner leaflet of the plasma membrane and form tubules on these liposomes [17,18]. Whether dynamin is able not only to form membrane tubules but also to constrict these tubules and cut membranes through its GTPase activity remained unclear [19].

While the drosophila genome contains only one dynamin gene, mammalian genomes have three dynamin genes named dynamin1–3. Dynamin1, the first identified and the closest to drosophila dynamin, is highly expressed in the brain, essentially in neurons, and very little in other tissues [13], although it is present in various cell lines where it plays a distinct role [20]. Dynamin2 is expressed ubiquitously and dynamin3 is also highly enriched in the brain, heart, lungs and testis [21]. Each dynamin isoform has 2–4 alternatively spliced sites, further expanding the diversity of dynamin isoforms [21].

All dynamin isoforms have a conserved domain structure (see Figure 3). Three domains were initially defined based on homology with known domains. First, the amino-terminal G domain is a GTPase domain with a structural homology to classical small Ras-like GTPases [22]. The intrinsic GTPase activity of dynamin is low but can be further enhanced by binding to microtubules or liposomes. This effect is due to dynamin oligomerization [23], suggesting an intermolecular interaction stimulating dynamin GTPase activity. Based on these homologies, many mutants with altered GTPase activity were generated [14,24,25]. Interestingly, the degree of modulation (decrease or increase) of GTPase activity correlates well with the level of CME assessed with internalization of transferrin, suggesting that this step is rate-limiting in the formation of endocytic vesicles [24–26].

Second, a pleckstrin homology domain (PHD) is located in the middle of the sequence. The PHD is able to bind anionic phospholipids such as PS and PIs (PI(3,4)P_2_, PI(4,5)P_2_ and PI(3,4,5)P_3_), albeit with low affinity [27]. Nevertheless, the dimerization of PHDs by fusion with glutathione-S-transferase domain enhances its apparent affinity [27,28] as it would occur with oligomerization of dynamin. Indeed, dynamin forms a tetramer in high salt conditions (resembling the physiological conditions) [29] and forms higher order, ring assemblies in low salt conditions [16]. Therefore, purified dynamin can bind liposomes and form tubules, depending on the ability of its PHD to bind lipids. Interestingly, dynamin without a PHD, or with a PHD unable to bind lipids, exerts a dominant negative effect on CME [30,31].

The third part of dynamin, which was best characterized was the C-terminal part called proline- and arginine-rich domain or PRD. The PRD is a major interaction hub for SH3 domain-containing endocytic accessory proteins [32], which bears several class I (RxxPxxP) and class II (PxxPxR) SH3-binding motifs (where P is proline, R is positively charged arginine and x is any amino acid) [33]. These motifs interact with various specificities to many SH3 domain containing proteins involved in CME such as intersectin1-2 [34], endophilinA1-3 [35], amphyphysin1-2 [36], syndapin1-3 [37], SNX9 and SNX18 [38]. The PRD is essential for the recruitment of dynamin to CCPs [39], and the dominant negative effect of dynamin GTPase mutant (K44A) is lost if the PRD is absent [33], suggesting an essential role of this domain in dynamin recruitment and function at CCPs. Moreover, expression of isolated SH3 domains of the aforementioned proteins competes with endogenous protein binding and blocks endocytosis at various stages of invagination [40–42]. The amphiphysin SH3 domain is the most effective at blocking endocytosis in neurons [40] and non-neuronal cells [42]. Similarly, intracellular injection of a peptide corresponding to the sequence of dynamin interacting with amphiphysin (QVPSRPNRAP [36], conserved in all three dynamins) blocks endocytosis as well [40]. This peptide, often referred as D15 peptide (a 15-mer issued from dynamin1), has been used extensively, mainly by electrophysiologists who use a patch-clamp recording pipette to dialyse the peptide into the cell cytosol to block dynamin-mediated endocytosis and follow its effects by measuring endocytosis directly [40,43–45] or its indirect effects on synaptic transmission or plasticity [46–50]. However, the D15 peptide has a low affinity and must be used at 1–2 mM for complete inhibition in neurons [45] but only partial inhibition in a fibroblast cell line [44]. To increase its effectiveness, we have designed dimeric peptides (dD15), which bind amphiphysin dimers with high affinity (amphiphysin forms oligomers at the neck of forming vesicles through its BAR (Bin Amphiphysin Rvs) domain [51]) and can fully block endocytosis [44]. Why is D15 apparently more effective in blocking endocytosis in neuronal presynapses than in non-neuronal cells? Neurons express and use mainly dynamin1 [52] while non-neuronal cells express mainly dynamin2 [53]. In addition to the identified amphiphysin SH3-binding site, dynamins bear additional type II SH3-binding sites, which can bind multiple SH3 domains simultaneously [44]. Dyn2 PRD bears one more SH3-binding site than Dyn1 PRD, which could contribute to the better targeting of dynamin2 compared with dynamin1 to CCPs in non-neuronal cells [54]. Therefore, D15 would compete more easily with Dyn1 than Dyn2 and block endocytosis. These differential effects reveal the multiple interactions of dynamins with its interacting partners, which can also be regulated by phosphorylation (see below).

The remaining domains of dynamin were termed middle domain (between the G domain and the PHD) and GTPase effector domain, or GED, because it was involved in the GTPase activity induced by oligomerization and could activate GTPase activity like an intramolecular GAP [26]. However, these domains did not bear obvious homology with known domains. Therefore, a complete structure of dynamin was needed to understand its function as a mechano-enzyme, but its tendency to form oligomers impaired early attempts to determine its full structure with crystallization.

## The next 20 years: structural and cellular studies

Thirty years after the characterization of the shibire^ts^ flies, at the beginning of 2000s, five major technical advances bolstered research on dynamin mechanism of action in cells: (i) live cell imaging of fluorescently tagged dynamin and associated proteins, (ii) the generation of dynamin knock-out (KO) mouse and cell lines, (iii) the development of small molecule specific inhibitors of dynamin function, (iv) structural determinations with X-ray crystallography and cryo-electron microscopy, and (v) quantitative assays of dynamin membrane binding and remodelling in minimal reconstituted systems. I will now review these developments and how they changed our view on dynamin function in cells.

## Live cell fluorescence imaging of dynamin function during endocytosis

In a pioneering study on the dynamics of proteins within cells, Gaidarov and colleagues [55] used expression of clathrin light chain (CLC) fused to GFP to observe clathrin-coated structures (CCS) on the surface of cultured cells. Consistent with the localization of CLC–GFP to endocytic pits, these CCSs appear and disappear at the surface of cells with detectable but minimal lateral movement during their lifetime [55]. Therefore, appearance of a CCS would correspond to the early clustering of clathrin and associated proteins while its disappearance would correspond to the final steps of vesicle formation, invagination, scission, movement of the endocytic vesicle and clathrin uncoating. Quantitative measures of CCS lifetime would thus provide a measure on how fast endocytic vesicles form, hence endocytic activity, now measurable in single cells or even subcellular structures, as opposed to classical uptake assays which require many cells or which are limited to a single time point. Indeed, a large number of studies have used this assay and developed sophisticated algorithms to detect, track CCSs and classify them according to their lifetimes [56–58], and found a link between block of endocytosis and stalling of CCSs, in particular interfering with dynamin function [59,60]. However, in most cell types, CCSs stabilize on the adhering face of cells after a few hours on the substrate, giving rise to more complex and stable CCSs called clathrin ‘plaques’, as opposed to small CCSs likely corresponding to individual coated pits [61–67]. The structure of these larger CCSs is difficult to resolve with conventional, or even total internal reflection fluorescence (TIRF) microscopy, which has a lateral resolution of approximately 250 nm, limited by the diffraction of light, that is lower than the average CCS size of 150–300 nm [68,69]. Structured illumination microscopy (SIM) with high numerical aperture objective (NA 1.65), which can achieve a resolution of 84 nm, combined with TIRF (which has an evanescent field decaying e-fold in 50 nm, enabling the resolution of very small vertical movements [70]), could resolve the predicted ring shape of individual CCPs (as cross-section of a dome structure) and showed that large and stable CCSs are formed of a single patch of fluorescence from which ‘multiple CCPs emerged like bubbles’ [71]. This is consistent with the observation that large CCSs produce vesicles detected with a direct method such as the pulsed pH protocol (see below [72]). To avoid the complication of dealing with these stable CCSs, most studies on molecular mechanisms of CME have used freshly plated cells to more directly relate the analysis of CCS lifetime with endocytic activity.

**Figure 2:**
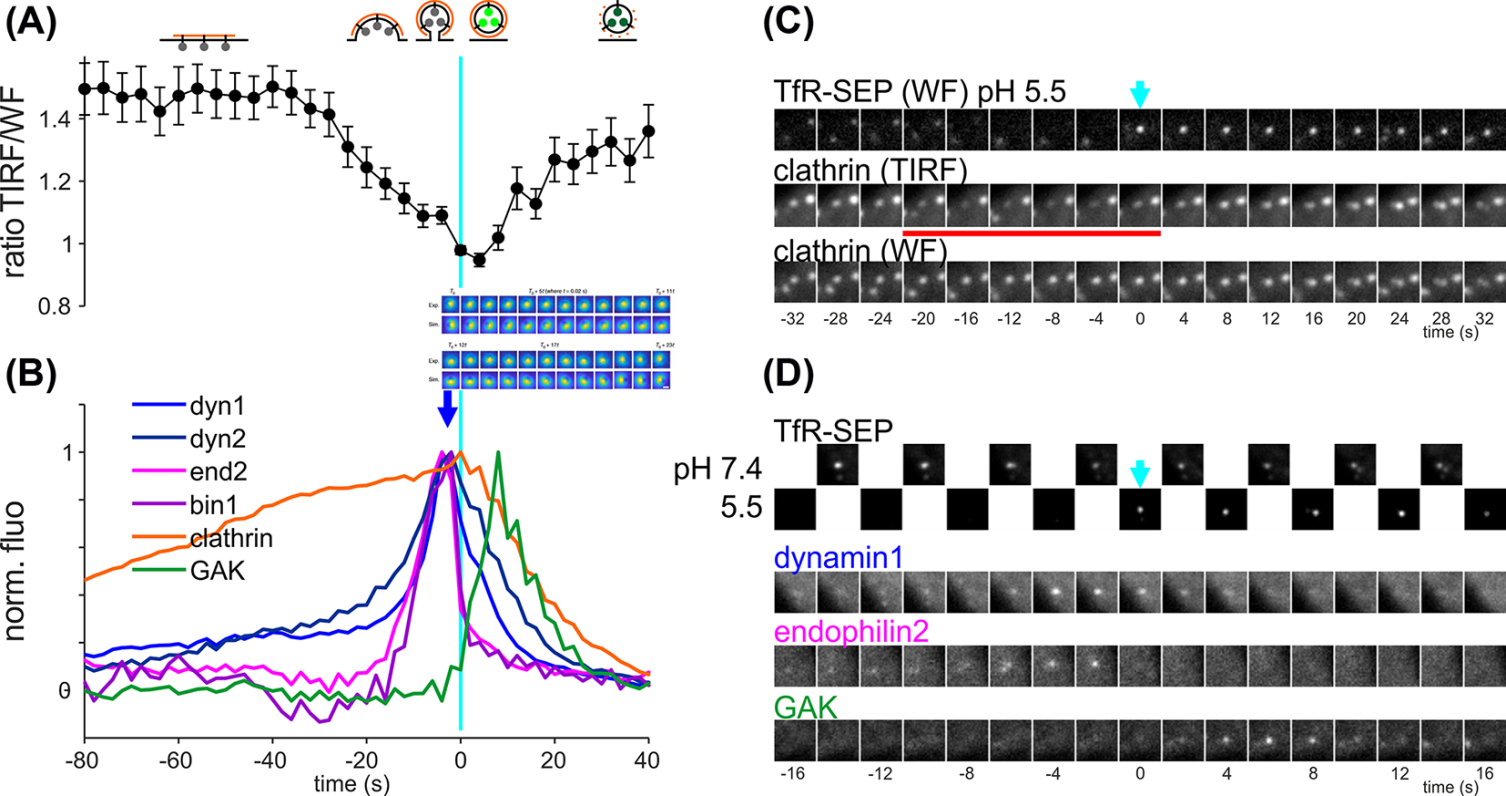
Recruitment of dynamin in living cells during CCV formation (**A,B**) Alignment of fluorescent traces to the time of CCV detection (time 0) determined with the ppH assay. (A) Ratio of fluorescence emitted by CLC-RFP excited by TIRF illumination (*z* length constant of *d* = 50 nm obtained with a 1.65 NA objective) and wide field (WF) epifluorescent illumination. Re-plotted from [72]. (B) Overlay of normalized clathrin light chain, dynamin1, dynamin2, endophilin2, bin1 and GAK fluorescence relative to time 0 (cyan line). Note that maximum dyn1/2 and endo2/bin1 fluorescence occurs at time -2 s, at the time of scission (i.e., 2–4 s before detection at time 0, when pH 5.5 solution is applied to detect CCVs). At this time, GAK fluorescence starts to rise. Endophilin2 drops abruptly at time 0 while dynamin slowly decreases in the next 20 s. GAK fluorescence peaks at 8 s. Re-plotted from [78]. Inset, 3D-SPORT imaging of single nanorod at high speed (50 Hz) showing a large rotation at peak dynamin recruitment. Reprinted from [87] (**C**) Example of TfR fluorescence at pH 7.4 and 5.5 with CCV detection (arrow), with the corresponding CLC-RFP illuminated with TIRF or WF. With TIRF illumination, the fluorescence of the central cluster clearly decreases 20 s before CCV detection (red line), while it remains constant with WF illumination, signalling vertical movement during membrane invagination. (**D**) Example of TfR-SEP fluorescence at pH 7.4 and 5.5 during TIRF illumination (*z* length constant of 120 nm), with the corresponding dyn1-mCherry fluorescence. Two more examples of endophilin2 and GAK fluorescence show the transient recruitment of these proteins at different times relative to scission. Reprinted from [78].

The addition to the microscopy toolbox of fluorescent proteins that can be distinguished from GFP, mainly ‘red’ fluorescent proteins, enabled the detection of proteins associated to clathrin during CME. One of the first candidates to test was obviously dynamin: by co-expressing dynamin1–GFP with CLC-DsRed, [73] could observe the recruitment of dynamin to CCSs. Of note, DsRed is a bright red fluorescent protein which unfortunately forms tetramers, strongly affecting the trafficking of most fusion proteins; nevertheless, CLC forms a very stable complex with clathrin heavy chain trimers, which explains the relatively minimal disruption of its trafficking by DsRed. Focusing on the disappearance of CCSs observed with TIRF microscopy, Merrifield et al. showed the transient recruitment of dynamin1–GFP reaching a maximum approximately 10 s before the start of CCS disappearance. This observation is consistent with the role of dynamin in vesicle scission. However, as pointed out immediately after this publication, the disappearance of the CCS from the TIRF field could reflect either departure of the endocytic vesicle after its formation, involving dynamin in the last step of vesicle scission or CCS invagination, involving dynamin in earlier steps [74]. One way to address this difficulty is to determine the recruitment of proteins at steps following scission. After scission, clathrin uncoating is mediated by the ATPase Hsc70 and its cofactor auxilin1 and auxilin2, also called cyclin G-associated kinase (GAK), so auxilin/GAK recruitment should occur after scission. Indeed, auxilin1–GFP or GAK–GFP is recruited just before the decrease of CLC-tomato signal, and its maximum recruitment follows dynamin1 maximum by a few seconds [75]. However, the definition of specific steps in the formation of endocytic vesicles still relies on the recruitment profile of a reference protein and is based on the known roles of this protein, which can be debated, as in the case of auxilins [76,77]. Another possibility to disentangle steps in the formation of endocytic vesicles is to detect the fission step directly. This was done by Merrifield and colleagues [72] with the so-called pulsed pH protocol (ppH), which monitors the location of a membrane cargo repetitively by extracellular application of low pH (5.5) buffer every 2 s (Figure 2). Cells transfected with the transferrin receptor (TfR), which is internalized constitutively and selectively through CME, tagged with the pH sensitive mutant of GFP superecliptic pHluorin (SEP), have a punctuate distribution in clusters co-localized with CCSs. However, if most TfR-SEP clusters disappear in low pH buffer, a subset resists quenching, indicating that they correspond to endocytic vesicles (clathrin-coated vesicles, CCVs). Then, with a slower time course, vesicles disappear as they move away, acidify or fuse with endosomes. By alternating between pH 7.4 and pH 5.5 every 2 s, the formation of CCVs can thus be simply detected as the sudden appearance of spots in images taken at pH 5.5 at pre-existing CCSs seen at pH 7.4. Thanks to this protocol, the time and location of single CCV formation can be precisely determined. How is this signature related to the classical ‘CCS disappearance’ signature for CCV formation? While some CCV detections, i.e. scission events, correspond to CCS disappearance, about half of them in Swiss 3T3 fibroblast cells are not associated with a detectable change in CCS fluorescence [72,78]. This latter type, termed ‘non-terminal’ event, could correspond to CCVs emerging from a stable structure, such as a plaque. In mature cultured neurons (>15 days *in vitro*), in which CCSs are distributed in dendrites and spines and are very static [62,79], nearly all scission events are categorized as terminal [80]. Therefore, the ppH protocol and its adaptations [80] is the only method to assess the endocytic activity in cells bearing static CCSs. In addition, a direct comparison between scission and CCS disappearance (half maximal decrease) shows a lag of ∼7 s but with a jitter (standard deviation) of 22 s between the two measures [78], which calls for caution in the interpretation of live cell imaging of CCSs and associated proteins [81]. However, despite the large interest in imaging CCS dynamics, the ppH protocol has been used by only a handful of laboratories [82–85], likely because it requires a fast perfusion system which would be difficult to build and adapt to conventional light microscopes, especially in common imaging platforms. We have published recently a detailed protocol, which will hopefully help diffuse this technique [86].

The vertical movements corresponding to coated pit invagination could be measured precisely with TIRF microscopy with a small penetration depth (50 nm) and alternate TIRF/wide field epifluorescence to normalize on the amount of clathrin at the CCS (Figure 2A,C). The TIRF/WF ratio is initially constant and starts to decrease 40 s before scission and reaches a minimum shortly after scission [72]. This decrease corresponds to CCS invagination and a general vertical movement of about 40 nm. Indeed, this movement prior to scission is corroborated by complementary measures of surface invagination with ion conductance microscopy [88] or plasma membrane orientation with polarized TIRF [89]. After scission, the TIRF/WF ratio increases again as the amount of clathrin decreases. This apparent movement could be due to the asymmetric uncoating of the vesicle, starting at the pole further away from the plasma membrane, or to a movement of the vesicle towards the plasma membrane after the vesicle neck, coated with dynamin and associated proteins, has disappeared [90].

What about dynamin recruitment during CCP invagination and vesicle formation? Taylor and colleagues [78] could show that indeed, dynamin1 or dynamin2 fused to mCherry are recruited maximally at the 2 s preceding detection of a CCV, which corresponds to the time of scission (Figure 2B,D). Moreover, GAK-mCherry is detected with a peak recruitment following dynamin recruitment by 10 s. Importantly, GAK-mCherry recruitment is significant only after the vesicle is detected [78], consistent with a specific recruitment of GAK after scission. Overall, the temporal precision of the ppH assay (2 s) is higher than the characteristic recruitment time course of most proteins associated with CCSs, enabling a quantitative study of these time courses and the definition of protein modules involved in CME [78,91], as was done before for CME in *Saccharomyces cerevisiae* yeast cells, which have more stereotyped endocytic events [92]. Remarkably, the N-BAR domain containing proteins amphiphysin2/BIN1 and endophilin2, which bind dynamin through their SH3 domains, are also recruited transiently with a maximum recruitment at -2 s, like dynamin1/2 [78]. While the initial kinetics of recruitment are similar before the peak, these proteins quickly move away from the site of scission after CCV formation (Figure 2B,D). This could be due to the disappearance of the vesicle neck after scission, which forms a template for N-BAR domain binding [51]. Alternatively, N-BAR proteins, although necessary for dynamin recruitment to CCSs, could impair scission [93], such that they must be released for scission to proceed.

In all cases, dynamin was consistently observed to be recruited in a burst of about 20 s shortly before CCS disappearance in cells expressing dynamin-GFP/mCherry after transient transfection [44,78,85,94–97], in stable cell lines [75,98] or in genome edited cells [56,99,100]. Before this transient burst, dynamin is recruited at lower levels in earlier steps of CCS maturation (Figure 2B) [56,78,98,99]. The degree of early dynamin recruitment may be higher for dynamin2 than that for dynamin1 [94,96], but see [78]. Moreover, the degree of early recruitment is higher in cells overexpressing mutants of dynamin1 with reduced GTPase activity, and this degree is correlated with the effect of the mutation [98]. Similarly, the rate at which dynamin is recruited in the late phase is lower with higher degree of inhibition of GTPase activity [98]. These results are consistent with a model in which the GTPase activity of dynamin contributes to its recruitment at late stages of CCS maturation, perhaps by participating in the constriction of the vesicle neck. Interestingly, dynamin2–GFP is also detected in CCSs visualized by platinum replica electron microscopy (PREM) [69]. This technique permits to visualize the intracellular side of the plasma membrane of adhering cells and interacting proteins. CCSs can be easily visualized with the characteristic hexagonal lattice of clathrin harbouring various degrees of curvature and lattice rearrangements [68]. When combined with super-resolution stochastic optical reconstruction microscopy (STORM) of anti-GFP nanobody labelled with Alexafluor 647, dynamin is present not only in highly curved CCSs but also in flat CCSs. As expected, dynamin2–GFP is concentrated in highly curved CCSs, which is consistent with its accumulation in a collar around the neck of the forming vesicle. In flat CCSs, dynamin is predominantly localized at its edge, together with FCHO2 and Eps15, proteins involved in the initiation of CCSs [101]. The fact that dynamin is present at low levels in flat/early CCSs could be passive, reflecting the binding of dynamin to interactors present at early stages, such as intersectin [34,101]. In addition, dynamin could play a role in the initial steps of CCS maturation, and act as a control checkpoint for vesicle formation [102].

## Blocking dynamin action with gene KO and chemical blockers

The essential role of dynamin in endocytosis was further demonstrated by the generation of KO mice and cell lines. The first published results concerned Dyn1^−/−^ KO mice [52]. As expected from the expression profile of the *dynamin 1* gene, the phenotype of Dyn1^−/−^ mice affects primarily neuronal cells. KO mice develop apparently normally *in utero*, but soon after birth, they present reduced milk absorption and poor motor coordination, leading to their premature death within 2 weeks. Electron microscopy of presynaptic terminals of cultured neurons revealed a large decrease in the number of pre-synaptic vesicles (SVs) and the appearance of aberrant structures formed by interconnected clathrin coated buds, which are accessible to the extracellular medium, suggesting an arrest of endocytosis at a late stage similar to the shibire^ts^ phenotype (Figure 1D). SVs are locally recycled after exocytosis, thereby blocking action potentials, which trigger most SV exocytosis, with tetrodotoxin reversed this phenotype. Importantly, live cell fluorescent imaging of SV exocytosis and recycling in neurons transfected with SynaptopHluorin (SypH) showed that indeed exocytosis is not affected, but that fast endocytosis, which occurs during a train of electrical stimulation in WT neurons, is totally blocked in Dyn1^−/−^ mice [52]. After electrical stimulation ceases, endocytosis resumes at a slow pace, presumably thanks to residual Dyn2/3 activity or endocytosis independent of dynamin. Re-expression of Dyn1 into KO neurons rescues the phenotype, as well as expression of Dyn3, which is also primarily expressed in the brain. However, re-expression of Dyn2 does not. Moreover, neuronal cultures prepared from double Dyn1^−/−^xDyn3^−/−^ mice have a similar, exacerbated phenotype compared with neurons from single Dyn1^−/−^ mice, while neurons from Dyn3^−/−^ mice have no apparent phenotype [9]. Therefore, Dyn1 and Dyn3 can mediate SV endocytosis, with Dyn1 having an essential role and Dyn3 are more accessory role. Importantly, the PRD domains of Dyn1 and 3 bind similarly to their main interactors, amphiphysin 1 and 2, SNX9, syndapin1 and endophilin1, whereas Dyn2 PRD interacts more weakly, which could explain the specific role dynamin 1/3 in SV endocytosis [9]. In addition, Dyn3 PRD binds the post-synaptic protein Homer1, which may play a role in locating CCSs near the postsynaptic density and affect the localisation and trafficking of post-synaptic glutamate receptors [103]. However, the post-synaptic localization and role of Dyn3 remains debated [9]. During electrical activity and SV exocytosis, Dyn1 is still able to perform endocytosis, and may even be activated by the large increase in cytosolic calcium which occurs during electrical activity. Indeed, dynamin was recognized early on as a dephosphin, a group of phosphoproteins, which are dephosphorylated by the Ca-dependent phosphatase calcineurin in response to stimulation, and which comprises several other proteins involved in endocytosis [104]. In neurons, Dyn1 is phosphorylated by Cdk5 [105] as well as GSK3β [106]. Quantitative phospho-proteomics on stimulated brain synaptosomes has revealed two patches of serine and threonine residues that are dephosphorylated in a calcium-dependent manner [107]. The first patch of five residues sits next to an SH3 domain binding site which binds mainly endophilin and syndapin, and phosphorylation of S774 and S778 in particular regulate syndapin binding and, to a lesser extent, endophilin binding [108]. The second patch of phosphorylated residues is specific for the splice variant Dyn1a, close to the amphiphysin binding site. So far, no functional effect of phosphorylation of these residues has been described. Nevertheless, the Dyn1a splice variant seems to be the only capable of rescuing the deficits in synaptic vesicle endocytosis observed in Dyn1^−/−^xDyn3^−/−^ neuronal cultures [109]. Dyn1a–GFP is clustered next to synaptic boutons while Dyn1b–GFP remains diffuse within the axon. This clustering is absent in phosphomimetic (S774,778D) Dyn1a while it is conserved with the phospho-deficient (S774,778A) Dyn1a [109]. This is consistent with a more than 2-fold decrease in endocytosis rate when rescue is mediated by phosphor-deficient Dyn1a compared with phosphor-mimetic Dyn1a [110]. On the other hand, Dyn1b contains a calcineurin-binding site, which is necessary for the internalization of the neuronal growth factor receptor TrkA and axon growth of sympathetic neurons, which is also regulated by the level of phosphorylation of S774 and S778 [111]. Interestingly, Cdk5 and GSK3β also regulate Dyn1 phosphorylation in non-neuronal cells and regulate CME [20] and fast endophilin-mediated endocytosis (FEME) [112], a clathrin independent form of endocytosis which depends on endophilin and dynamin [113]. Intriguingly, FEME and ‘ultrafast’ SV endocytosis in neurons [114] share many molecular and functional links [115].

Given the central role of dynamin and the ubiquitous expression of Dyn2, it was not surprising to see that full KO of the dynamin2 gene results in embryonic lethality [53]. Therefore, conditional KO (dynamin 2^loxP/loxP^) were generated and primary fibroblasts were obtained. However, after recombination leading to dynamin2 KO, dynamin1 expression was increased, leading to incomplete inhibition of CME, as found earlier in other dynamin2 KO cells [53,54]. Therefore, double conditional Dnm1^LoxP/LoxP^;Dnm2^LoxP/LoxP^;CRE-ER^+/0^ (DKO) cells were generated. In these DKO cells, and also in Dnm1^LoxP/LoxP^;Dnm2^LoxP/LoxP^; Dnm3^LoxP/LoxP^;CRE-ER^+/0^ (TKO) cells [8], CME is totally blocked, leading to a massive redistribution of the CME cargo TfR to the cell surface and the appearance of aberrant CCPs with long tubules and a single coated bud [53], reminiscent of the aberrant structures observed in Dnm1^−/−^ and Dnm1-/-xDnm3-/- neuronal cells [52,9], albeit without branching or vacuole-like pattern (Figure 1C). Strikingly, these aberrant tubular intermediates concentrate the endocytic protein endophilin 2 as well as a dense actin network, suggesting that endophilin and actin act upstream of dynamin function, even though the exact contribution of N-BAR containing proteins such as endophilins, amphiphysins and SNX9/18 in CME is a matter of debate [112,116,117]. Overall, the generation of dynamin KO mouse and cell lines demonstrate the central role of dynamins in many forms of endocytosis including SV endocytosis in neurons and CME, even though other forms of dynamin independent endocytosis can co-exist and compensate for their absence in neurons [118,119] and non-neuronal cell lines [53]. Moreover, dynamin TKO cells provide an important tool to study the effect of hypomorphic dynamin mutations on endocytosis [44,54].

To address the role of dynamin, and more generally the role of (dynamin dependent) endocytosis in cellular processes, considerable effort was dedicated to develop specific small molecule inhibitors of endocytosis. As described above, competing peptides such as the D15 peptides have been used extensively by neuroscientists but, because these peptides are not cell permeable, their use has been limited to studies with direct access to the cytoplasmic compartment, essentially patch-clamp electrophysiology. A myristoylated, cell permeable version of D15 has been used [44,46] and is commercially available. Early blockers of endocytosis, such as chlorpromazine, monodansylcadaverine or potassium depletion, lack specificity (see [120] for a comprehensive review). In 2006, a small molecule screen on dynamin GTPase activity identified dynasore as a non-competitive, cell permeable inhibitor of CME with minimal acute cell toxicity [60]. Dynasore quickly became a tool of choice to inhibit endocytosis in various cellular contexts [58,119,121–124]. However, dynasore is very sensitive to inhibition by serum and trace amounts of detergents. To alleviate these caveats, a less sensitive, more specific and potent version of dynasore was identified and called dyngo4a®, with an IC50 of 5.7 µM for inhibition of Tfn uptake [125,126]. Nevertheless, both dynasore and dyngo4a are not specific for dynamins: they also block the mitochondrial dynamin-related protein Drp1 [60] and block fluid-phase endocytosis and membrane ruffling in dynamin TKO cells [8]. Other dynamin inhibitors have been developed, targeting either the binding of PHD to membranes or directly the GTPase activity [120]. Among them, dynole 2-24 [127] shows the best combination of high potency (IC50 of 1.9 µM for inhibition of Tfn uptake), cell permeability and low cytotoxicity. However, a full characterization of its effects in various cellular contexts remains to be established. In conclusion, a better understanding of binding and mode of action of dynamin inhibitors should help their design and optimization. These inhibitors may have therapeutic value, in combination with other drugs, for example, to treat some cancers with immunotherapy [128,129].

## Structure of dynamin around lipid tubules

To understand how dynamin GTPase activity can lead to vesicle scission, it is crucial to understand how it forms oligomers around membrane tubules and deforms them after GTP binding and hydrolysis. Decisive understanding of this process was enabled by structural studies and biophysical assays (reviewed in [32, 130, 131]).

Dynamin oligomers wrapped around lipid tubules could be finally resolved with cryo-electron microsopy (cryo-EM) in their native state at a resolution of ∼20 Å [132]. At this resolution, T-shaped units could be ascribed to dimers of dynamin in a right-handed helix. The PHDs probably correspond to the lower part interacting with the lipid bilayer and the G domains forming the two upper tips of the T, likely interacting with each other in the same helix rung and with other neighboring rungs of the helix. In total, 13–15 units are required to form a turn of the helix, corresponding to 26–30 dynamin molecules. Dynamin lacking the PRD (dynΔPRD) could also form helices around tubule with similar properties but could also constrict the tubules with addition of the non-hydrolysable GTP analogues β-γ-methyleneguanosine 5′-triphosphate (GMP-PCP), β-γ-imidoguanosine 5′-triphosphate (GMP-PNP) or GTPγS. Full-length dynamin could not induce such constriction suggesting that the PRD opposes constriction when GTP hydrolysis is impaired. The remainder of the T-shaped unit was occupied by the rest of dynamin consisting of middle and GED domains, but their actual arrangement could not be resolved. Further processing of long helices by iterative averaging showed that helix constriction by GMP-PCP was mainly due to the contraction of the middle part [133]. However, the limited resolution of these early cryoEM studies precluded further characterization.

**Figure 3:**
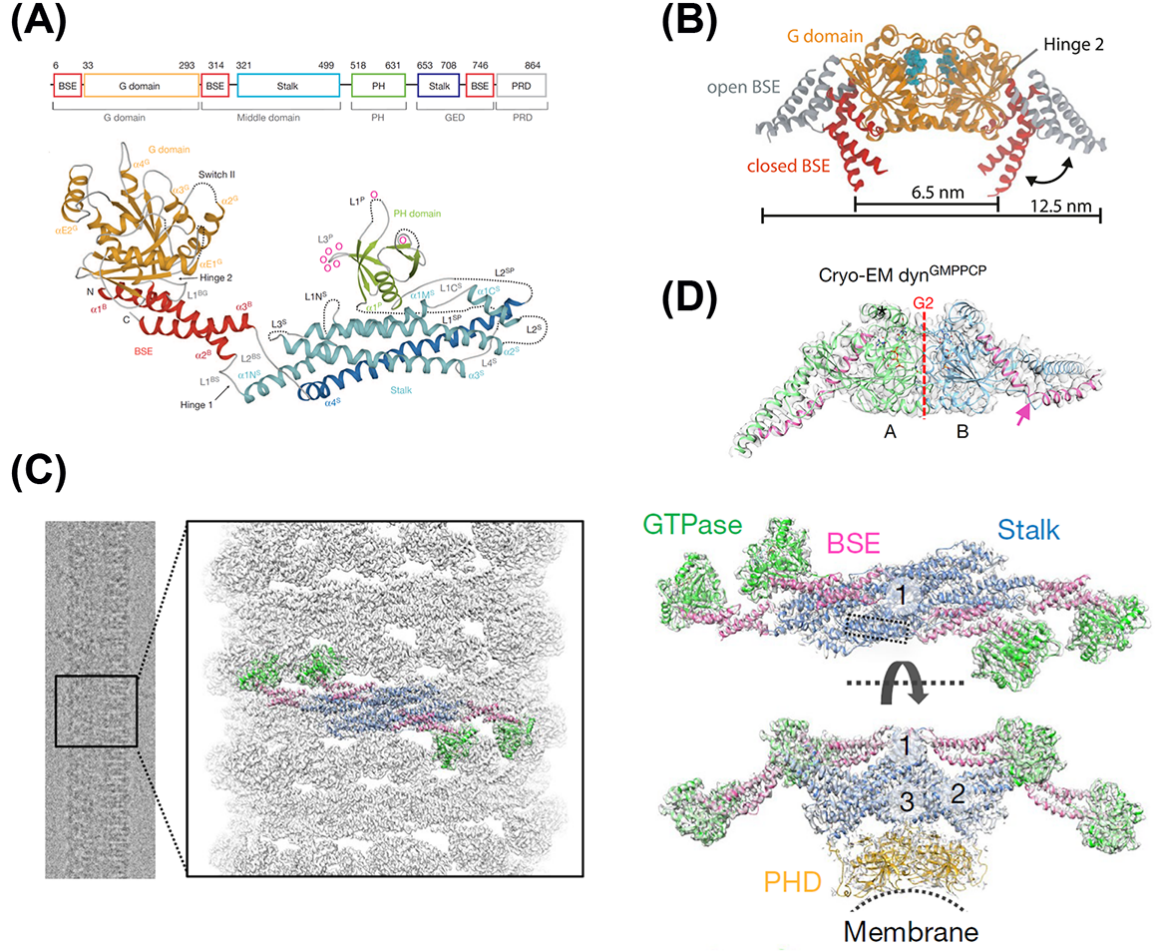
Structure and recruitment of dynamin to membrane tubules (**A**) Structure of nucleotide free human dynamin1. Top, structure-based domain architecture of human dynamin1. The classical domain assignment (with the old ‘middle’ and GED domains) is indicated below. Bottom, ribbon-type representation of human dynamin1 (PDB: 3SNH). Regions not resolved in the crystal structure are indicated by dotted lines. Domains, distinct secondary structure elements and N- and C-termini (connected to the PRD, absent) are labelled. Lipid-binding residues are indicated as o. Reprinted from [134]. (**B**) Overlay of open (GMP-PCP/GTP bound) and closed (GDP bound or nucleotide free) G-domain dimer crystal structures. Estimates for the distance between hinge 1 attachment points, are shown. Cyan spheres show the nucleotide-binding location. Reprinted from [144] (**C,D**) Cryo-EM map of assembled dynamin in the GTP-bound state (GMPPCP) on membrane at 3.75 Å. (**C**) Left, Cryo-EM images of helical dynamin tubes were processed to generate a 3D map, and subsequently a model of the tetramer was built (green, GTPase domain; pink, BSE; blue, stalk; gold, PHD) (Electron Microscopy Data Bank (EMDB) code: EMDB-7957; RCSB Protein Data Bank (PDB) ID: 6DLU). Right, tetramer model of assembled dynamin with surrounding density and domains coloured as described above. The assembly interfaces are labelled 1-3. Reprinted from [145]. (**D**) Asymmetry of MM domains in the cryo dynGMPPCP dimer reveals a unique kink (pink arrow) in the extended helix from the GTPase to the BSE (T274-E310, coloured pink) in the monomer labelled B (blue) but not in the monomer labelled A (green). Dashed red line indicates interface G2. Reprinted from [145].

Because of its propensity to oligomerize at high concentration, full-length dynamin resisted crystallization attempts. Nevertheless, two groups, Faelber et al. [134] and Ford et al. [135], succeeded at the same time in obtaining the crystal structure of a dyn1ΔPRD mutant, which does not oligomerize (IHGIR395-399AAAAA, K562E for [134] and G397D for [135]). This led to a re-assignment of the domain structure of dynamin (Figure 3A): while the GTPase and PHD were conserved and corresponded well to the structures of the isolated domains [22,136–138], the middle and GED ‘domains’ were reassigned as (i) a bundle signalling element (BSE) close to the GTPase domain composed of 3 α-helices and corresponding to 3 sequences located in N- and C-terminal of the GTPase domain and just before the PRD and (ii) a ‘stalk’ domain, a large elongated domain corresponding to two large stretches around the PHD sequence and composed of 4 α-helices and loops of various sizes in between. The crystals are composed of symmetrical dimers of dyn1ΔPRD with a large interaction interface between the stalk domains (called interface-2) assembling in a criss-cross fashion. This basic dimer structure is highly conserved along dynamin family members, such as the antiviral myxovirus resistance protein 1 (MxA) [139], the mitochondria fission protein Dynamin-like protein 1 (DNM1L, also called Drp1 [140]) and the mitochondrial genome maintenance 1 protein (Mgm1 [141]). Two additional interfaces between the stalk domains, interface-1 near the BSE and GTPase domains, and interface-3, in a loop at the other end of the stalk domain, where the disruptive mutations are located, were predicted from the crystal structure, but not observed directly, because the dimer forms a linear array different from the predicted helicoidal array of dynamin1 around lipid tubules (see Figure 3C, right). Solving the structure of a tetrameric dynamin with a less disruptive mutant of dynamin 3, dyn3ΔPRD(K361S), confirmed the existence of these two interfaces [142] with a dimer of non-symmetrical dimers. Docking of the crystal structure into the helical structure observed with cryo-EM predicts the dimerization of the G domains from the 10th dimer on the next turn (for a 13:1 helix). Indeed, the crystal structure of the G domain with its flanking domains almost corresponding to the BSE revealed a large dimerization interface of the G domains, which controls the GTPase activity of dynamin [143]. However, this dimer interaction is too weak to be significant in solution in isolated G+BSE, also called motor module (MM) domains [144]. Therefore, the increase in dynamin GTPase activity inducted by the dimer (interface-2) and higher order multimer formation into a helix around lipid tubules will lead to the possible dimerization of the G domains after at least one helix turn has been completed (Figure 3C).

The crystal structure of the MM domains with GMP-PCP (mimicking GTP) or with GDP.AlF_4_^−^ (mimicking GDP.P_i_ hydrolysis intermediate) revealed a large rotational movement (∼69°) of the BSE relative to the attachment to the G domain (Figure 3B), from an ‘open’ conformation to a ‘closed’ conformation [146]. The latter also corresponds to the crystal structure without nucleotides [134]. GTP hydrolysis could thus generate a large conformational change generating a power stroke which leads to membrane tubule constriction [130]. These transitions were recently measured in isolated G+BSE domains in solution with single molecule FRET, confirming the large movements observed in crystals [144]. Moreover, these measures together with measures of nucleotide binding rates and GTP hydrolysis rates of GG dimers enable the construction of a quantitative model of dynamin remodeling of membrane tubules with GTPase activity [144], which I will describe further after evoking the biophysical studies in the next paragraph.

Finally, recent high-resolution (3.75 Å) cryo-EM study of dyn1ΔPRD has validated the model constructed with crystallographic data, with binding through PHDs, dimerization of G domains across helical rungs, and revealing the predicted interfaces 1 and 3 (Figure 3C) [145]. However, the cryo-EM structure obtained with GMP-PCP has several important differences compared to the crystal structures. First, the position of the BSE relative to the G domain is different for one of the monomers because of a kink in the second BSE helix (Figure 3D), which suggests a strain in the dynamin pushing on the membrane, which may provide an additional force leading to constriction. Indeed, mutations inducing a tightening of this alpha helix and a disruption of the kink strongly inhibit CME [145]. Second, the PHDs were poorly resolved, reflecting a large flexibility of the link between the stalk and the PHDs, also found in the various crystal structures, where the location of PHDs are quite variable [134,135,142]. This flexibility would ensure adaptation of the dynamin binding to variable membrane tubule diameters.

## Seeing dynamin in action in reconstituted *in vitro* systems

Early *in vitro* assays of dynamin incubated with lipids and various GTP analogs concentrated on EM studies and, because they required fixation (cryo or chemical), rely on static observations. However, it is also possible to follow with high-resolution differential interference contrast (DIC) microscopy the formation of lipid tubules coated with purified brain dynamin [147]. Remarkably, addition of GTP induces a stiffening of these tubules and their eventual scission if they are attached at both ends of the substrate. Tubules with a free end twist and supercoil, which suggests that dynamin GTPase activity induces a twisting of the dynamin assembly, as predicted by the structural studies [18]. To measure this twisting motion, streptavidin-coated polystyrene beads, which bind to biotinylated dynamin, were added. Rotations of these beads around the tubules could be clearly detected and the frequency of these rotations is a function of GTP concentration with a half maximal speed approximately 400 µM. Remarkably, this value is close to physiological GTP concentrations, suggesting that GTP supply could be a modulator of dynamin function [148]. Interestingly, this twisting motion could be recently detected in living cells with imaging gold nanorods (80 nm length, 40 nm section) coated with transferrin (Tfn-NR) [87]. Single Tfn-NR localize on the cell surface together with CLC-mCherry and dynamin2–GFP. Tfn-NR orientation can be monitored at high speed (50 Hz) by 3D single particle orientation and rotational tracking (3D-SPORT). Around the peak dynamin2–GFP accumulation, a fast and large rotational movement of the Tfn-NR occurs (130 ± 56° angle in 0.28 ± 0.18 s in 45 observations) (Figure 2B)[87]. This cargo movement probably reflects the twisting motion occurring while dynamin hydrolyses GTP, as it was not observed in cells overexpressing the GTPase inactive mutant dynamin2 K44A.

**Figure 4:**
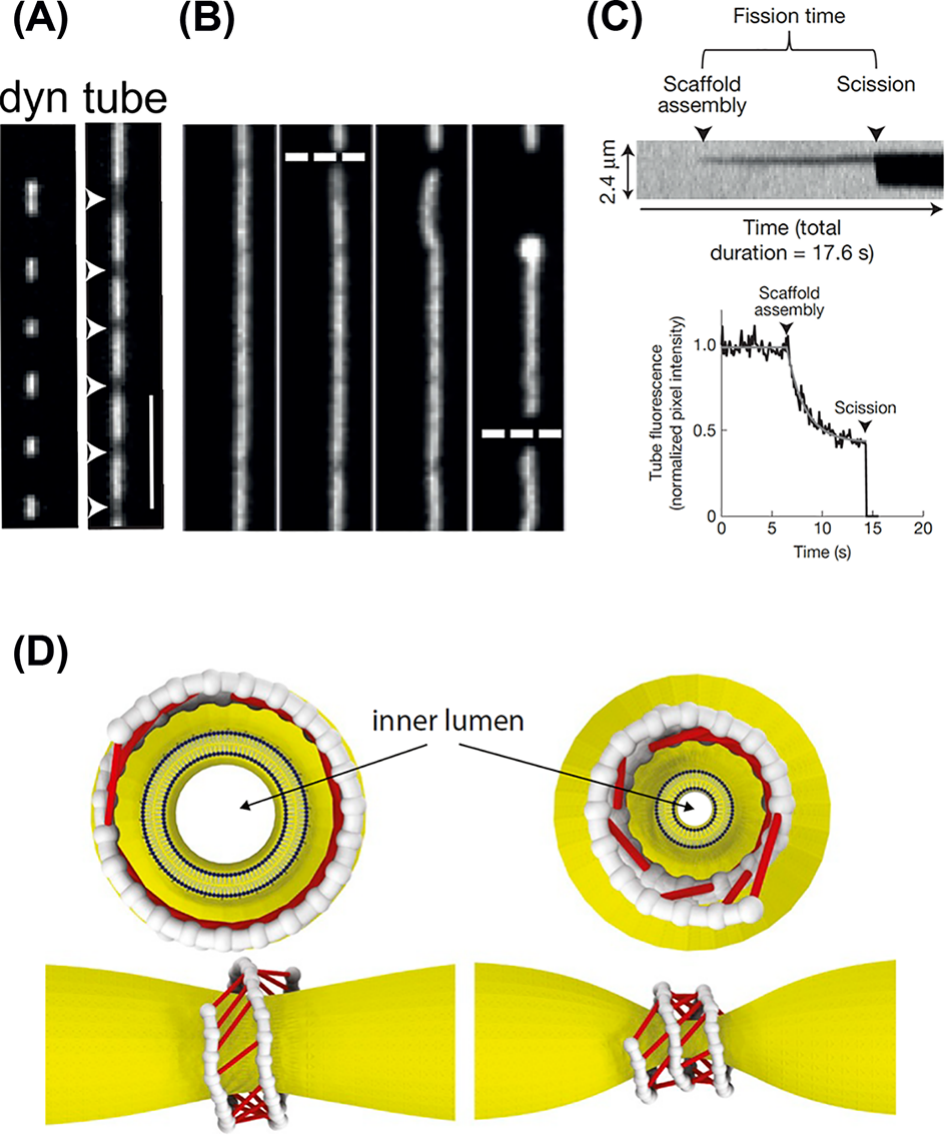
Scission of membrane tubules by GTPase activity of dynamin *in vitro* (**A–C**) Recruitment of dynamin1 (labelled with Alexa488) to preformed membrane tubules (labelled with p-Texas red DHPE) and scission with GTP. (A) Recruitment of dynamin without GTP. At the sites of dynamin recruitment, tubule fluorescence is reduced which corresponds to sites of tube constriction (white arrowheads); scale bar: 5 μm. (**B**) in the presence of GTP, scission quickly follows constriction. Images of tubules at 10 s time intervals. (**C**) Top, a representative kymograph from experiments as in (B) for a single tube scission event. Bottom, single-pixel fluorescence trace from the kymograph shown on top showing kinetics of tube constriction before scission, fitted to an exponential decay function (average time constant 6.5 s). Reprinted from [153]. (**D**) A model of dynamin constriction developed by Ganichkin and colleagues [144]. Snapshots of the initial (left) and final (right) steady states for a filament with *N* = 40 dimers and duty ratio ω = 0.75. Cross-bridges are indicated as red links. The tube is constricted from inner lumen radius of 10.5 to 2.5 nm.

In early *in vitro* studies of dynamin function and in all structural assays, dynamin is incubated at high concentration (∼10 µM); hence, its ability to induce membrane tubules. However, at lower concentration, dynamin binds preferentially preformed small diameter membrane tubules. Tubule diameter can be controlled by pulling membrane from a giant unilamellar vesicle with a bead manipulated with optical tweezers with controlled surface tension [149,150], by pulling nanotubes from planar lipid bilayer with a glass pipette [151,152] or by extrusion by controlled buffer flow and calibrated measures [153]. In these conditions, dynamin acts as a sensor of membrane curvature, which binds preferentially tubules with a small radius (approximately 18 nm in [150]). This effect was visible for a concentration of dynamin above 280 nM, below the estimated concentration of 400 nM in cells. Dynamin, visualized with fluorescence, initially binds to discrete patches of tubules, which act as seeds for further growth, which occurs steadily at a rate of 14 nm/s [150]. This speed amounts to about one helix rung (12 nm) per second. By measuring the amount of dynamin and the lipid tube radius with fluorescence, Morlot and colleagues [149] and Dar and colleagues [153] could clearly detect tube constriction which occurred for a critical external lipid tube radius below 16 nm. Constriction reduces the tube radius to approximately 12 nm, close to the predicted value from structural studies [132,145]. Addition of GTP induces further constriction and eventually scission when the tube radius reaches 7.3 nm [153], which is agreement with estimates based on conductance measurements [151]. In the constant presence of GTP, addition of dynamin led to constriction and scission within approximately 20 s [149,151,153], and this time was dependent on GTP concentration (Figure 4A–C). The rate of constriction increased with GTP such that it became kinetically inseparable from scission and that the length of dynamin scaffold (and hence the number of dynamin molecules) cannot be deduced from these experiments. Nevertheless, estimates of specific hydrolysis rate of dynamin in the presence of lipids (>4 s^−1^) predict that 18 steps of GTP hydrolysis may occur during this time span.

Recently, a model of dynamin GTPase cycle leading to membrane constriction was developed [144], based on the biophysical characterization described above and further measures of GTP hydrolysis and conformational changes of the G+BSE motor module (MM) and the force developed by one power stroke (Figure 4D). This model predicts that a short filament of 40 dynamin dimers with a probability of interacting G domains (or duty ratio) above 0.5 can lead to constriction of the tubule below 4 nm radius, at which scission may occur, within seconds. In addition, the rate of constriction can be greatly accelerated if the G dimer dissociation depends on the strain exerted on it. Constriction would occur through sliding of adjacent helix turns, as predicted and observed with high speed atomic force microscopy [154]. Moreover, this model predicts that for long dynamin filaments, such as the ones observed in *in vitro* studies when dynamin and GTP are added sequentially, constriction occurs primarily at the edge of these filaments. This could explain why scission of tubules is observed near the edge of dynamin clusters [149,155] (but see [153]). Nevertheless, in cells, dynamin would start constricting as early as few GG dimers are permitted across helix rungs.

Finally, how does dynamin GTPase activity produce scission? It was originally suggested that constriction of tubules is sufficient to lead to spontaneous scission. Nevertheless, the PHD is not only responsible for dynamin binding to membranes but could also play an active role in scission. The variable loop 1 of the PHD (L1^P^ in Figure 3A) inserts into the lipid membrane [156] and mutating the large hydrophobic isoleucine residue 533 in cysteine or alanine does not abolish dynamin binding to preformed PIP_2_ containing tubules [153,157] but expression of dyn1 I533A in cells blocks CME [157]. Therefore, membrane insertion of the PHD may catalyze scission, even if it is not absolutely required for tubule scission [158].

## Counting the number of dynamin molecules at sites of scission

The biophysical and structural studies of purified dynamin with lipids predict that tubule constriction and fission can occur as dynamin dimers wrap around the tubule and G dimers can dimerize to activate dynamin GTPase activity, which will take place with a minimum of 13 dimers. Is this situation encountered in the more complex cellular environment?

In cell lines in which both alleles of the dynamin2 gene have been edited to bear a GFP tag, all Dyn2 molecules are fluorescent, enabling the quantification of the number of proteins at sites of scission. Using the signal emitted by single GFP molecules adsorbed on glass and identified by single step photobleaching, the signal at the peak can be converted in number of Dyn2 molecules. In human SKMEL–Dyn2–GFP cells, there were 35 ± 13 molecules at the peak [159] and in human SUM–Dyn2–GFP cells 50 ± 19 molecules at the peak (or 33 ± 14 if starting 10 s before the peak, at the end of the steady phase) [99]. These estimates are remarkably similar given the fact that they rely on two different cell lines and two different types of fluorescence microscopes (TIRF vs spinning disk microscopes). This number corresponds to a little more than one predicted turn around a membrane tubule (or vesicle neck), which takes 13.3 dimers of dynamin (i.e., 26-27 Dyn2–GFP molecules), with 25% between 26 and 40 molecules (1–1.5 turns), 27% between 40 and 52 molecules (1.5–2 turns) and almost half more than 52 molecules. However, several factors could lead to an underestimate of the number of dynamin2 molecules at scission. First, not all GFP molecules are necessarily fluorescent because they have not matured properly [160] or because they have bleached. Using single GFPs immobilized on glass for calibration uses by construction only fluorescent molecules. Second, in the case of TIRF microscopy, but also for any fluorescence microscope, the position of the GFP on the glass coverslip would increase its brightness compared with GFP molecules within a cell, separated from the glass interface by at least a few tens of nm, due to near field effect for fluorescence emitted close to the glass interface [70]. Third, the signal of a single fluorophore is much smaller than the combined fluorescence of 50+ molecules, so even though detection systems such as EMCCD cameras are built and calibrated as linear, departures from linearity would greatly affect the coefficient for converting fluorescent values into number of molecules. To mitigate these caveats, calibration of fluorescence can be achieved by expressing in cells self-assembling nanocages labelled with GFP with a known number of subunits (from 12 to 120, in the range of the expected number of Dyn2–GFP molecules) [161]. With this method, Akamatsu et al. estimated that ∼200 copies of the Arp2/3 complex protein ArpC3-GFP (in genome edited cells) are recruited at CCSs during endocytic vesicle formation. The Arp2/3 complex nucleates actin filaments, with important consequences on key cellular functions including endocytosis [116,162]. This method could lead to a new estimate of the number of dynamin molecules at the peak of its recruitment and at the time of scission.

Can the degree of dynamin recruitment at scission be modulated? As previously discussed, actin plays a variable but important role in facilitating CME. Indeed, incubation of latrunculin-B, which binds to actin monomers and thus inhibits the formation of actin filaments, reduces endocytic activity by 50% in NIH 3T3 fibroblast cells [72,98]. As expected, actin markers are no longer recruited to forming CCVs as detected by the ppH assay, but more unexpectedly, the amount of dynamin recruited at the time of scission is also reduced by more than 60%. Moreover, endophilin2 recruitment is completely abolished, but the post-scission recruitment of GAK is preserved, which shows that the scission events detected still correspond to forming CCVs [98]. Therefore, vesicle scission can occur, albeit less efficiently, with a reduced number of dynamin molecules. Similarly, in dynamin TKO cells with partial rescue of CME by a dynamin2 mutant (dynamin2 R830A, R833A) fused to mCherry, the peak dynamin fluorescence at the time of scission is reduced by half [44]. These two examples show that dynamin recruitment can occur at variable levels during endocytic vesicle formation, and that it is a major determinant of the efficiency of CME.

## Conclusions and perspectives

After 50 years of research on endocytosis and focus on one of its mediators, dynamin, we have reached a detailed understanding of its role during endocytic vesicle formation. In this review, I have focussed mainly on CME and synaptic vesicle endocytosis, but dynamin is involved in many other forms of vesicle scission, including clathrin independent forms of endocytosis such as FEME [113], reticulon3-dependent endocytosis [163], endocytosis induced by extracellular ligands such as shiga toxin [124], phagocytosis, scission following dense core granule exocytosis, or kiss-and-run [164], as well as intracellular vesicle scission. On the other hand, dynamin is not involved in all forms of endocytosis, in particular in yeast cells which lack a dynamin gene even though they undergo CME, which shares many molecular mechanisms with CME in mammalian cells [116]. More generally, membrane scission can be achieved by a variety of molecular mechanisms which do not necessarily involve an active mechanism, such as GTP hydrolysis in the case of dynamin [165].

Dynamin has a well-documented role in vesicle scission, but it also regulates cellular structures enriched in filamentous actin, such as podosomes, phagocytic cups or lamellipodia (reviewed in [166]). Recently, dynamin was involved in the formation of filamentous actin enriched podosome-like structures in Drosophila myoblast fusion [167]. Dynamin acts by bundling actin filaments around dynamin rings via an actin–PRD interaction. Dynamin GTPase activity disassembles the rings and the releases the attached actin filaments, enabling Arp2/3-mediated generation of branched actin filaments, which enables myoblast fusion [167]. Interestingly, the mutations in the PRD which disrupt actin-PRD binding are the same as the ones which disrupt amphiphysin SH3-PRD binding and thus regulate CME [44]. Consequently, dynamin roles in CME and actin bundling may compete with each other. On the other hand, the formation of actin filaments regulates CME [116]. It would be important to address the direct role of dynamin in initiating actin filament bundling in CME.

Finally, dynamin2 is mutated in autosomal dominant centronuclear myopathy (ADCNM), a muscle disease characterized by defects in organelle positioning in myofibers, and some forms of Charcot-Marie-Tooth peripheral neuropathies. Interestingly, in the reported cases encompassing more than 100 families, mutations have been found exclusively in the PHD and stalk domain [168]. The effect of these mutations on dynamin function remains unclear, but their dominant character most likely reflects a gain of function. This is consistent with the fact that dynamin2 overexpression in myofibers leads to an ADCNM-like phenotype in mice [169]. BIN1, which interacts with dynamin, is also linked to some ADCNM cases. Moreover, it recruits dynamin2 to membrane tubules and regulates tubules fission and its overexpression improves muscle atrophy in dynamin2 ADCNM mouse models [170], linking these two scission proteins in a complex phenotype. Whether or not this phenotype is directly linked to scission activity remains to be established.

Dynamin has become a prototypical mechano-enzyme, which is involved in a large range of cellular processes and best characterized in CME. With the development of more accurate tools and assays to analyse its structure and function in cellular processes, understanding the mode of action of dynamin and its regulation by cellular factors will remain a major question in cell physiology for years to come.

## Abbreviations

ADCNM: autosomal dominant centronuclear myopathy
CCP: clathrin-coated pit
CCS: clathrin-coated structure
CCV: clathrin-coated vesicle
Cdk5: cyclin-dependent kinase 5
CME: clathrin-mediated endocytosis
CNM: centronuclear myopathy
FEME: fast endophilin mediated endocytosis
FRET: Foerster Resonance Energy Transfer
GAK: cyclin G dependent kinase
GED: GTPase effector domain
GSK3β: glycogen synthase kinase 3 isozyme β (there are no GSK1 or 2)
PHD: pleckstrin homology domain
PI: phosphatidylinositol
ppH: pulsed pH protocol
PRD: proline- and arginine-rich domain
PREM: platinum replica electron microscopy
PS: phosphatidylserine
SH3: Src homology domain type 3
STORM: stochastic optical reconstruction microscopy
SypH: synaptophluorin
Tfn: transferrin
TfR: transferrin receptor
TIRF: total internal reflection fluorescence microscopy
